# Pivoting to Childbirth at Home or in Freestanding Birth Centers^1^ in the US During COVID-19: Safety, Economics and Logistics

**DOI:** 10.3389/fsoc.2021.618210

**Published:** 2021-03-26

**Authors:** Betty-Anne Daviss, David A. Anderson, Kenneth C. Johnson

**Affiliations:** ^1^ The Pauline Jewett Institute of Women’s and Gender Studies, Faculty of Arts and Social Sciences, Carleton University, Ottawa, ON, Canada; ^2^ Centre College, Danville, KY, United States; ^3^ School of Epidemiology and Public Health, Faculty of Medicine, University of Ottawa, Ottawa, ON, Canada

**Keywords:** COVID-19, cost effectiveness of homebirth, safety of homebirth, ACOG statements on homebirth, freesstanding birth centers, medical intervention, out-of-hospital birth

## Abstract

Birth-related decisions principally center on safety; giving birth during a pandemic brings safety challenges to a new level, especially when choosing the birth setting. Amid the COVID-19 crisis, the concurrent work furloughs, business failures, and mounting public and private debt have made prudent expenditures an inescapable second concern. This article examines the intersections of safety, economic efficiency, insurance, liability and birthing persons’ needs that have become critical as the pandemic has ravaged bodies and economies around the world. Those interests, and the challenges and solutions discussed in this article, remain important even in less troubled times. Our economic analysis suggests that having an additional 10% of deliveries take place in private homes or freestanding birth centers could save almost $11 billion per year in the United States without compromising safety.

## Introduction: Trying to Stay at Home for Everything During COVID: Why Would You Risk Going Anywhere Else for Childbirth?

**FIGURE 1:**
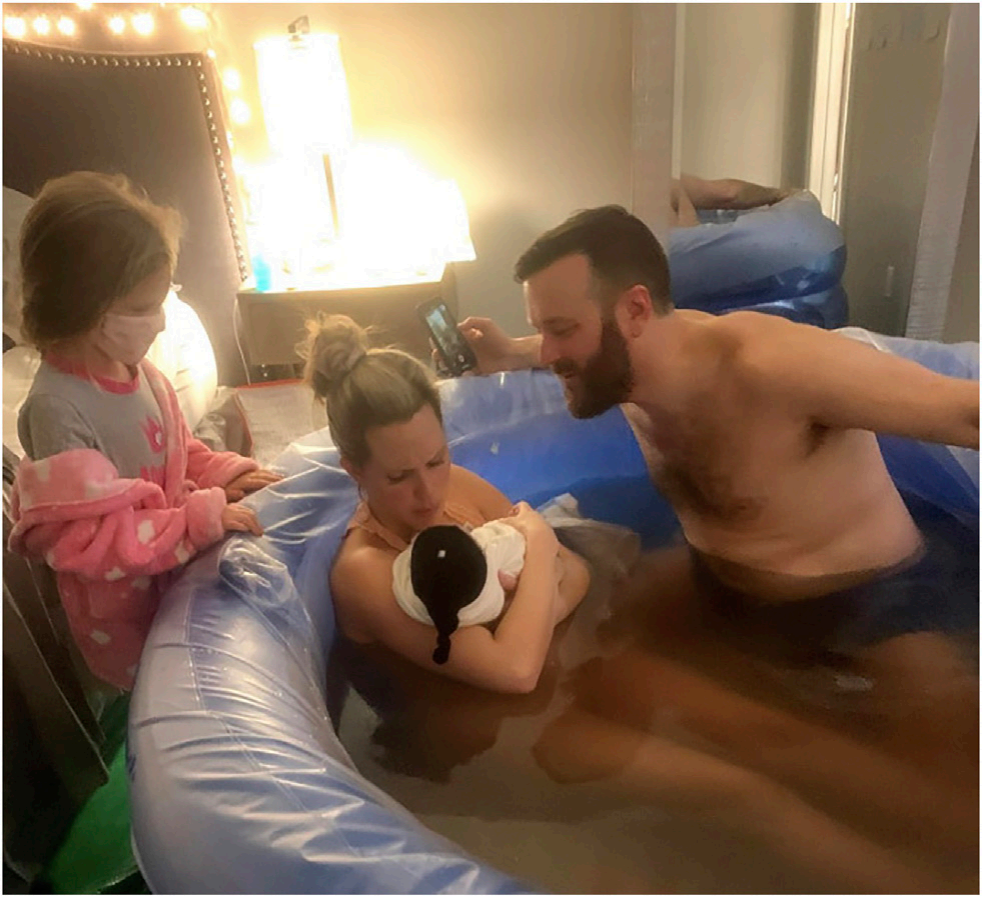
Home birth in the time of COVID-19: Millennial father and lawyer, Robert Onley, who caught his own son in the pool in their master bedroom, puts aside his mask and iPhone momentarily, while midwives stand back for both photo-op and physical distancing and the father's real-time moment with the new baby. Midwife protocol is that the mother, Natasha Onley can birth without a mask. Daughter, Isabelle, stands by watching, still with her mask on, for the benefit of the midwives, who have to do births in other settings, and are therefore careful themselves as well to use Personal Protective Equipment (PPE). Photo by grandmother, Lori Szauter. Used with permission.

**FIGURE 2:**
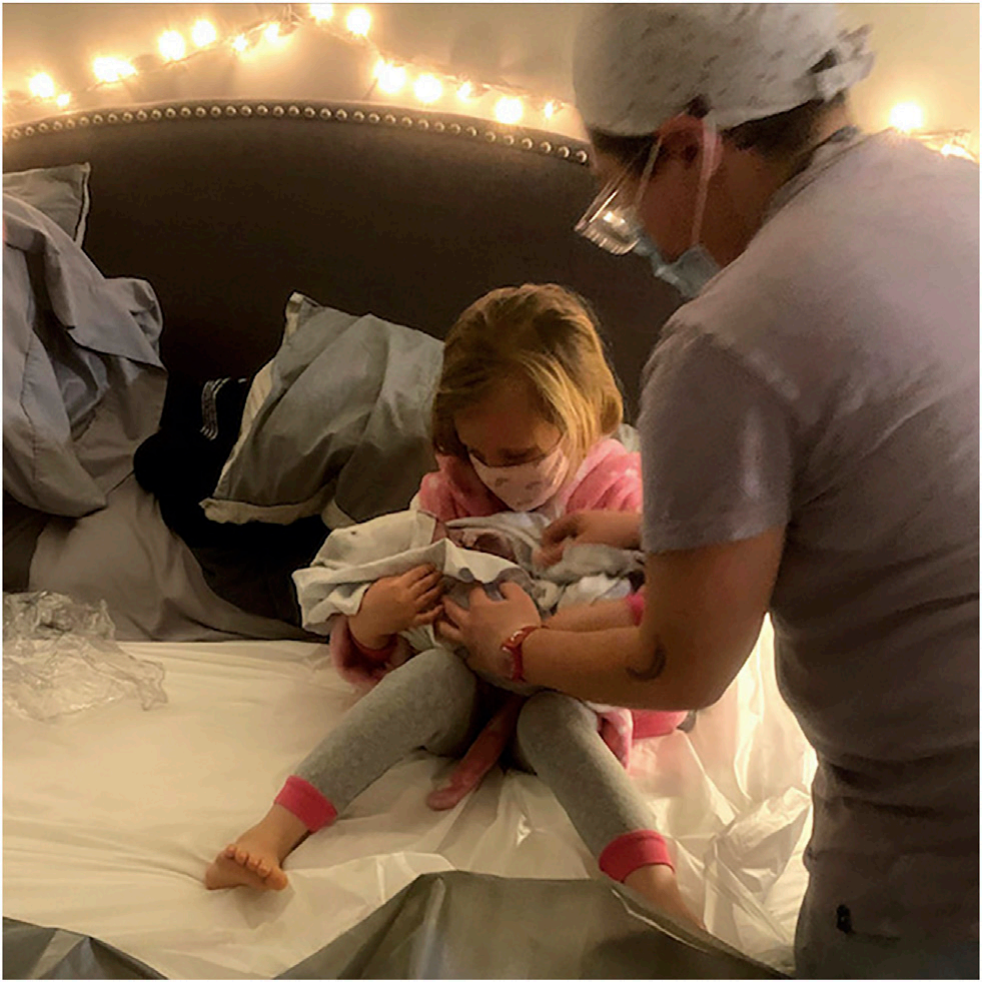
Isabelle, age 5, one of the few children who will never ask “Where do babies come from?” cradles her new little brother, shortly after he comes out of the water. Midwife Ness Dixon, helping her, has already had both doses of the Pfizer vaccine, but both American and Canadian midwives continue to maintain caution, encouraging family members to wear masks, whether the baby is born at home or in hospital. Photo by Lorie Szauter. Used with permission.

**FIGURE 3:**
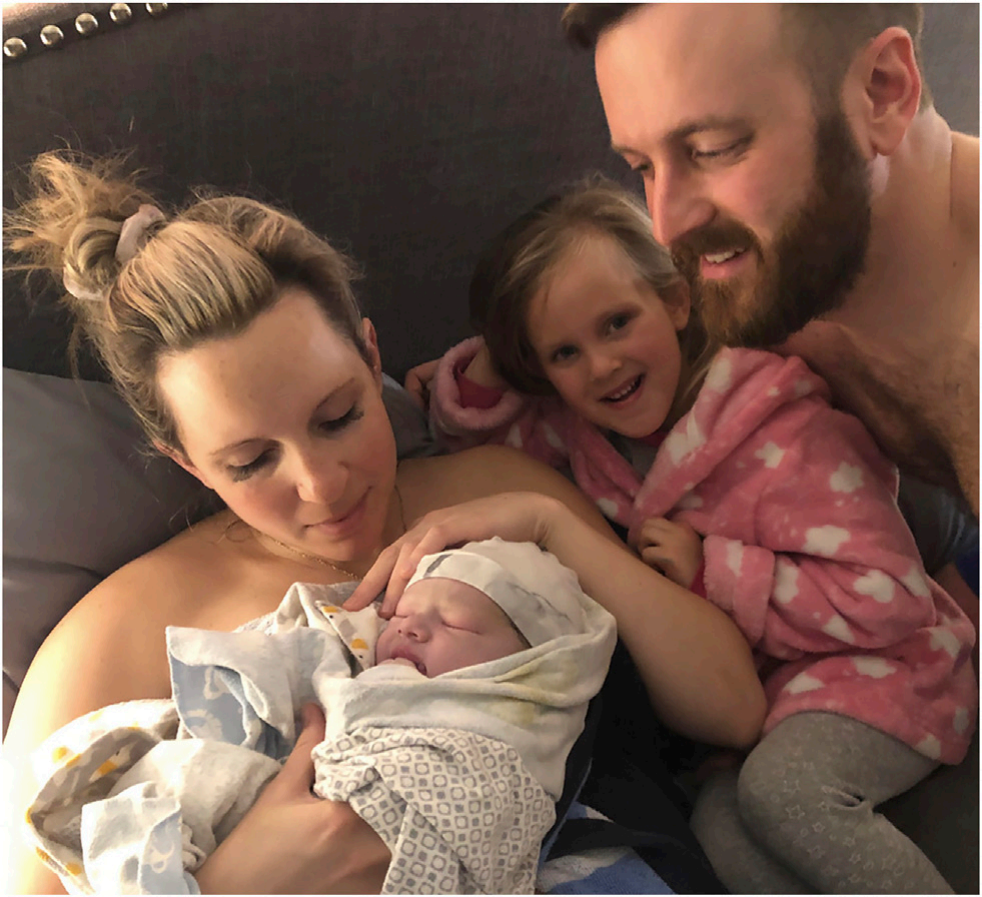
The family gathers together in the family bed. In Canada, all births–home, hospital, or birth center–are covered through government insurance. Families can choose where they want to deliver, unhampered by considerations of cost. Midwives stand back again while the family is afforded a photo without masks, taken by grandmother, Lori Szauter. Used with permission.

**FIGURE 4:**
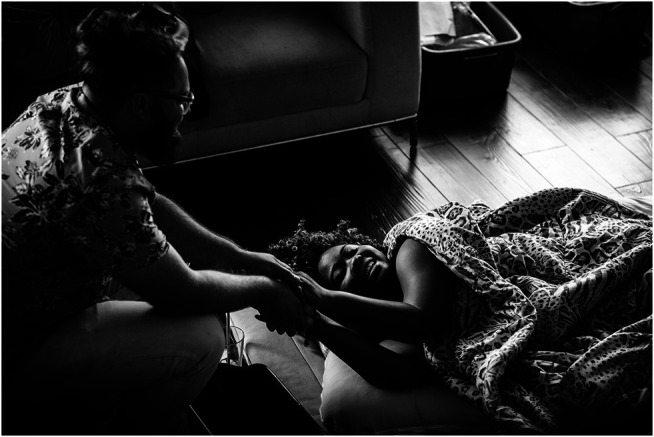
Nicholas Richer-Brulé holds the hands of his wife, Bernadette Betchi, during a contraction. They chose a home birth because “it is a safe place where we were able to deliver our baby in the comfort of an environment that we could control. This meant even more with the unpredictability that Covid-19 has had on our surroundings. It eliminated the stresses of traveling while in labor, of being separated from each other and our children and being subjected to the hospital's restrictions and rules” (personal communication, Bernadette). Photo by Elle Odyn Breathe In Photography Ottawa Ontario. Used with permission.

Births at home or in a freestanding birth center were increasing in the US even before COVID-19, but since decisions around birth generally center on safety, giving birth during this pandemic has brought safety challenges to a new level. As hospitals began to apply COVID restrictions, increasing numbers of childbearers made the decision to be supported during labor by their partners in their private homes (See Figures 1–4), instead of facing birth alone in hospitals–in the very buildings that take in the people who are sickest with this new plague (Davis-Floyd et al., 2020). While these personal safety threats to laboring people have relaxed in many areas to allow at least the partner into the hospital, and in spite of the vaccine being rolled out, it is not likely that other restrictions in hospitals, or the dangers, are going to disappear anytime soon.

Furthermore, amid the COVID-19 crisis, the concurrent work furloughs, business failures, and mounting public and private debt have made unnecessary personal and community/state expenditures an inescapable concern. For years, maternity and newborn care have constituted the largest hospital payouts from commercial insurers and state Medicaid programs, and the per-capita expenditures in the United States exceed those in every other high-resource country (Truven Health Analytics, 2013). Before COVID-19, the Committee on Assessing Health Outcomes by Birth Settings of the National Academies of Sciences, Engineering, and Medicine (NASEM, 2020: vii) clearly stated, to anyone still unaware at the beginning of 2020: “The United States spends more on childbirth than any other country in the world, with worse outcomes than other high-resource countries, and even worse outcomes for women of color.”

As we will detail in this article, birthing persons have been continually achieving safe outcomes in private homes and freestanding birth centers with the assistance of midwives in the United States and abroad. Even so, there has been reluctance to include all nationally credentialed midwives in publicly funded US maternity care programs and state licensure policies. Resistance stems from beliefs that home or freestanding birth center births are riskier than hospital births^2^.

COVID-19 has disrupted the perspective of actual safety because staying at home offers better protection from the pandemic for childbearers than sharing a hospital with disease-stricken patients. While freestanding birth centers, unlike hospitals, are not the settings where COVID-19 positive individuals go for treatment, they still present the risk of contamination from other patients, staff, and visitors. Yet as at hospitals, practitioners providing care in private homes and freestanding birth centers can take safety measures that include masks, sanitizing measures, and a minimized number of people at the birth (Figure 1–2), as other articles in this Special Issue demonstrate.

The economic analysis of public policy is usually a struggle with trade-offs. Consider a policy that increased the speed limit. It would save time, the trade-off being a predictable increase in traffic fatalities and carbon emissions. Yet in this article, we demonstrate how a public policy that expanded midwifery in the United States could save billions of dollars *without* necessitating trade-offs regarding safety. This is the first study to estimate the specific savings from public policy that increases births in private homes or freestanding birth centers by a given percentage. We intend to demonstrate that greater access to maternity care by credentialed and licensed midwives in these settings is a solution that is safe, cost effective, and increasingly popular.

For practical models, we can draw on the experiences of countries that have invested in publicly funded home and freestanding birth center births. For example, starting in the 1980s, the Canadian provincial governments charged lawyers and consultants to research a birth model that was safe, cost effective, and met the needs that childbearers were asking for. The solution: to give midwives legislative support and require the provision of a range of birth settings. Almost all provinces have implemented midwifery legislation since it was established in the province of Ontario in 1993. Now 11% of Canadian births are attended by midwives, and in the two provinces with the most midwives—B.C. and Ontario—25 and 15% of births respectively are under midwifery care (Canadian Association of Midwives, 2019). Midwives in Canada in almost all jurisdictions are required by their Colleges (their regulatory bodies) to provide both home and hospital births paid for through universal not-for-profit government agencies (Figure 3).

Two major breakthroughs in the last four years have occurred suggesting that former opponents to home birth and to the use of a specific group of midwives, Certified Professional Midwives (CPMs) may have softened their views:

(1) The statements on home birth during the last four years by the American College of Obstetricians and Gynecologists (ACOG, 2016) have acknowledged women’s right to choose and agreed that home birth is safe in countries with well-integrated midwifery systems;
(2) Faced with the pandemic, an emergency Executive Order by Governor Cuomo of New York State permitted midwives licensed in other states or Canadian provinces, including Certified Professional Midwives, who had long been illegal in New York, to practice legally there for the initial period of major outbreak in the state (Executive Order #202.11). The timeline has continued to be extended^3^.

To be clear, Certified Professional Midwives (CPMs) are the only US midwives whose educational standards require them to undergo specialized clinical training in private homes or freestanding birth centers as a condition of national certification. They are also the only US midwives who are not allowed to practice in hospitals, and they can practice legally in only 36 states, with legislation pending in others.

The pressing questions now are: Will the gaps in the US maternity care system, and the solutions generated during COVID-19 be recognized as important when the pandemic is gone? Will increasing the numbers of midwives trained to work in private homes and freestanding birth settings and fully integrating them into that system during COVID-19 finally be recognized as a paradigm shift that will serve birthing people in normal times?

In what follows, we examine the intersection of the *safety* and *economic efficiency* of birth in private homes and freestanding birth centers, which has become even more critical as the coronavirus ravages bodies and economies around the world. We contend that those interests, and the solutions of increased legislation, liability insurance, and better integration for midwives working in those settings remain important even in less troubled times.

### The Pre-COVID-19 Increase in Home Births and Freestanding Birth Centers in the US

After a gradual decline from 1990 to 2004, the number of out-of-hospital births in the US increased from 35,578 in 2004 to 62,228 in 2017, so that 1 of every 62 births took place in homes and freestanding birth centers (1.61%) (Macdorman and Declercq, 2019). By 2015, there were more home births in the United States than in any other industrialized country (Martin et al., 2017)^4^.

Who is available to provide births outside the hospital in the US? Certified Nurse-Midwives (CNMs) attend births primarily in hospitals; in 2018, 9,399—only 2.6% of the births that they attended were in private homes and 11,139 (5.1%) in freestanding birth centers (Martin et al., 2019). Medicaid care is mandatory in all states and most Medicaid programs reimburse CNMs at 100% of physicians’ rates. The majority of states also mandate private insurance reimbursement for CNM/CM services (American College of Nurse-Midwives (ACNM), 2019).

In 2018, CPMs and other midwives who are not CNMs^5^ attended 16,823 (55.7%) of their births in private homes and 7,127 (23.6%) in freestanding births centers. Clearly these groups specialize in birth in the larger community outside the hospital. Again, CPMs rarely—if ever—have hospital privileges. CPMs are not currently recognized under Medicaid at the federal level. However, as of December 2020, 14 of the states in which CPMs are legal have also opted, through a state plan amendment, to cover CPM services^6^. CPMs and families who want access to their services are seeking federal recognition to secure Medicaid coverage in all states in which CPMs are licensed and meet certain educational requirements^7^.

It is important to emphasize that births attended in private homes and freestanding birth centers require providers specifically trained to do so with proper equipment, protocols in place for transport to hospital, and back up hospitals pre-arranged. As one physician reports:

I have served as a collaborative physician for several CNMs making the transition from hospital to home birth practice and have seen how steep the learning curve is, especially in their first year. To focus on safety in home and birth center birth, then we have to admit that it requires a different skill set than hospital birth and that providers practicing in the community setting must be trained in that skill set to maintain the safety of the environment (Personal communication, Sarita Bennett, DO, CPM).

Although many Americans have assumed that more CNMs could start doing home births if they so desired, it appears difficult for the US administrative facilities to consider something the other way around--that CPMs could work in hospitals. Because Canada deliberately chose not to create distinctions between nurse-midwives and other midwives at legislation, it is rare that Registered Midwives in Canada are also nurses. Yet all midwives in the standard Canadian model must have hospital privileges and do at least some hospital births, as well as home births.

In Canada, in the US states that have legislated and adopted insurance coverage for CPMs, and in other countries that have discovered or continued to recognize the importance of midwives who provide care in the community outside the hospital, a critical commonality has emerged. Bringing these midwives out from underground economies to have them fully integrated into what the World Health Organizations calls “the Reproductive, Maternal, Newborn and Child Health (RMNCH) Continuum of Care^8^,” secures the creative strategies most adaptable and safest for families of that community, not just for pandemics but for normal times.

In the US in 2018, midwives attended 10.2% of births (Martin et al., 2019), with a home birth rate of <2%. There are no data yet available to establish how much home births and freestanding birth center births are on the rise with COVID-19, but there is ample suggestive evidence from across the country that it is: in professional journals (see Davis-Floyd et al., 2020; The Trust Project, 2020, and other articles in this Special Issue), and in a substantial increase in news media coverage about midwives^9^ and the increasing numbers of US families who are seeking to give birth with midwives outside the hospital. One website called “Birth Monopoly” helps consumers track hospital policies to decide which one might have the least restrictions or whether the family feels secure enough to allow the laboring mother to go in at all^10^. Thus, investigating the efficacy and feasibility of better integrating and increasing birth in alternative settings seems timely.

## Evidence of Safety: Outcomes of Birth in Hospital vs. in Private Homes and Freestanding Birth Centers

The two most recent meta-analyses examining perinatal outcomes for birthing people with low-risk pregnancies in high-income countries have demonstrated similar levels of safety for hospital and planned, midwife-attended births in private homes or freestanding birth centers. An Australian meta-analysis (Scarf et al., 2018) found no significant difference in the odds of intrapartum stillbirth or early neonatal death (0–7 days), regardless of whether the birth was planned for home, birth center, or hospital, and no difference in those odds between parous and multiparous women. That meta-analysis of four studies of planned home births also identified significantly lower odds of NICU admission than for planned hospital births, with an odds ratio (OR) of 0.71 and a 95% CI of 0.55–0.92. Scarf et al. (2018) concluded that their findings “support the expansion of birth center and home birth options for women with low-risk pregnancies.”

A 2019 Canadian meta-analysis found 14 eligible international studies—representing more than 500,000 home births—which met their strict criteria for comparing planned home to planned low-risk hospital birth (Hutton et al., 2019). Stratifying their analyses by whether or not the midwives attending the home births were well integrated into the health services, they found that in jurisdictions where midwives were well integrated, perinatal and neonatal mortality summary risk estimates were essentially identical for intended home births and intended hospital births. The summary OR was 1.07 (95% CI, 0.70–1.65) for primips and 1.08 (95% CI, 0.84–1.38) for multiparous women.

In less integrated settings, Hutton et al. (2019) found that there was a possible increase in perinatal and neonatal mortality with home birth compared to hospital birth. However, because both estimates had large confidence limits due to the small numbers of deaths on which they were based, chance cannot be ruled out for the increase—the estimate on primips was based on 1 newborn death in 897 home births (The estimate for primips was OR 3.17 (95% CI, 0.73–13.76), and for multips, 1.58 (95% CI, 0.50–5.03).

Despite limited institutional support for credentialed midwives in the United States attending births in private homes and freestanding birth centers, the weight of evidence in US cohort studies indicates that births in these settings have good outcomes when the studies: 1) are based on charts rather than birth certificates, because the latter often lack accurate outcome and care details; 2) identified low-risk women; 3) are able to discern the planned place of birth, thereby avoiding counting accidental, unplanned out-of-hospital births; and 4) are conducted on a defined group of midwives with training standards. Where comparisons are possible, these US cohort studies (Murphy and Fullerton, 1998; Schlenzka, 1999; Johnson and Daviss 2005a; Stapleton et al., 2013), produced similar results for low-risk births at home, in birth centers or in hospitals, just as the international meta-analyses have found. Even where the defined group of practitioners had questionable homogeneity of education and a varying degree of integration into the US maternity care system, outcomes were similar to those in the other studies cited for low-risk birthing people (Cheyney et al., 2014).

## Evidence on the Costs of Hospital vs. Home and Freestanding Birth Centers

### Having the Safety for a Fraction of the Cost

This section demonstrates that births in homes and freestanding birth centers are far less expensive to society than hospital births. Combined with the evidence that outcomes are similar among low-risk mothers who plan their births in private homes, birth centers, or hospitals, this fact reveals a win-win situation: childbearers choosing their own home or a freestanding birth center can have the safety of hospital births at a fraction of the cost to families or insurers. The relevant discussion, then, is about whether the size of the “win” is worthwhile.

There are approximately 3.9 million births annually in the United States (Statista, 2019). The average charge by a midwife for an uncomplicated home birth is $2,870 (this and all costs are in 2019 inflation-adjusted US dollars (Anderson and Anderson, 1999). In freestanding birth centers, the average cost is $7,240 (American Association of Birth Centers, 2015). In hospitals, the average cost for an uncomplicated vaginal birth is $12,156 (Childbirth Connection, 2013).

Table 1 summarizes the potential savings from a modest increase in the use of private homes or freestanding birth centers in the United States. If an additional 5% of deliveries occurred in private homes rather than in a hospital, the savings would be $1.811 billion annually. If another 5% of deliveries occurred in freestanding birth centers rather than hospitals, the added savings would be $959 million annually. Note that about 10–20% of birthing people who plan to deliver at home or in a freestanding birth center transfer to a hospital during labor (Stapleton et al., 2013; Cheyney et al., 2014), so the number of planned out-of-hospital births would need to increase by about 6% in order for the actual increase to be 5%. For this analysis, we make the simplifying assumption that those transferred to hospital would pay the average costs associated with hospital births. Table 1 is reproduced from Anderson et al. (2021).

**TABLE 1:** Estimated birth costs and annual savings from an additional 10% of deliveries occurring in private homes or freestanding birth centers.

	Home birth	Birth center birth	Hospital birth	Savings from additional 10% home and freestanding birth center births (US dollars)
Estimated cost for an uncomplicated vaginal birth	$2,870^a^	$7,240^b^	$12,156^c^	
Additional 5% home births and additional 5% freestanding birth center births	$1.811 billion^d^	$959 million^e^		$2.769 billion
Lower cesarean rate for low-risk birthing people				$299 million^f^
Reduced rate of low birthweight babies				$111 million^g^
If competition brought 10% reduction in hospital birth cost				$4.267 billion^h^
Reducing cesarean rates in hospitals to 15% as WHO recommends (i)				$3.422 billion^j^
Total potential cost savings				$10.868 billion^k^

^a^ This figure is from Anderson and Anderson (1999), updated (as are all figures) to 2019 dollars using the Consumer Price Index. More recent studies of home birth costs are scarce and these costs vary widely by location. The cost for the midwife here is an estimate for the birth only, in order for it to be comparable to hospital birth. Midwives generally include prenatal and postpartum care in their fee, but this care is not included in this analysis for any of the birth locations.

^b^ This is the mean of the total of professional and facility charges for freestanding birth center births from the Practice Profile data collected from the Perinatal Data Registry by the American Association of Birth Centers (2015).

^c^ This is the average facility, labor, and birth charge for a vaginal hospital birth with no complications in 2011 (updated to 2019 dollars) as reported by Childbirth Connection (2013), obtained from the US Agency for Healthcare Research and Quality, available at http://hcupnet.ahrq.gov/. Published costs that are much lower than this represent a subset of the costs of birth, and perhaps only the cost of the hospital stay itself.

^d^ Calculated as 3.9 million births × 0.05 × ($12,156 - $2,870).

^e^ Calculated as 3.9 million births × 0.05 × ($12,156 - $7,240).

^f^ Low risk was defined as singleton, head-down term babies when data were obtained from the NVSS system to do the calculations for the “CPM 2000” study (Johnson and Daviss, 2005a). The savings from lowering the cesarean rate were calculated as [3.9 million × 0.05 × (0.19–0.052) × $5,735] + [3.9 million × 0.05 × (0.19–0.061) × $5,735].

^g^ Calculated as 3.9 million × 0.10 × (0.024–0.011) × $21,876.

^h^ Calculated as 3.51 million × 0.10 × $12,156.

^i^ See http://www.who.int/reproductivehealth/publications/maternal_perinatal_health/csstatement/en/.

^j^ Calculated as 3.51 million × (0.32–0.15) × $5,735.

^k^ Calculated as $1.811 billion + $959 million + $299 million + $111 million + $4.267 billion + $3.422 billion.

### Cesareans, Instrumental Deliveries, and Other Interventions: High Costs and Risks

In the Scarf meta-analysis (2018), women planning a hospital birth were nearly three times as likely to have a cesarean or instrumental (forceps or vacuum) delivery as those planning a home birth, and nearly twice as likely to have a cesarean as those planning a birth center birth. Similarly, there has been consensus across the literature for decades that planned home and birth center births in the United States entail significantly less medical intervention than planned hospital births (Johnson and Daviss 2005a; Cheyney et al., 2014; Hutton et al., 2019).

Our cost analysis of interventions focuses on cesareans because they are both the costliest intervention and the cause of numerous safety concerns. Cesareans are associated with a two-fold increase in maternal mortality, increased maternal blood loss, impaired neonatal respiratory function, increased incidence of maternal postpartum infections, increased fetal lacerations, trouble with maternal-infant interaction, extended length of stay and recovery, re-hospitalization, placenta accreta and previa, hysterectomies, transfusions of ≥4 units, maternal ICU admission, and uterine rupture (Spong, 2015). It is beyond our scope here to quantify the economic costs of a current cesarean on future pregnancies.

Although the risk of a serious problem during a typical cesarean birth is low, with almost one-third of US births being cesareans, problems occur and costs are high. The cesarean rate for planned hospital births in the United States is 32% (Martin et al., 2018), compared to 6.1% for planned birth center births (Stapleton et al., 2013) and 5.2% for planned home births (Cheyney et al., 2014). While some of the hospital births involve higher-risk childbearers with increased needs for cesareans, the majority of those cesareans are performed on those who were low-risk, begging the question, “Were they necessary?” To illustrate, data obtained from the National Vital Statistics System suggest that in 2000, when the overall US cesarean rate was 22.9%, low-risk women delivering in a hospital had a 19% cesarean rate, compared to a 3.7% rate for women who planned home deliveries with Certified Professional Midwives (Johnson and Daviss, 2005a).

A cesarean adds an average of $5,735 to the cost of a birth in the United States (International Federation of Health Plans, 2016). With the reduced likelihood of cesareans among the additional 5% home deliveries and the 5% birth center deliveries in our proposal, even if low-risk women still had only a 19% cesarean rate in hospital, the savings for families or insurance companies would be an additional $299 million annually.

### The Costs of Low Birth Weight and Prematurity

When prenatal care is provided by credentialed midwives, the incidence of low birthweight decreases. For example, the rate decreased from 2.4 to 1.1% in a national study (Johnson and Daviss, 2005b) and from 2.8 to 1.8% in a study conducted in Washington State (Health Management Associates, 2007). As well, the premature birth rate at the National Institutes of Health (NIH) for non-Hispanic white births in hospital has been shown to be more than double the rate for clients cared for by Certified Professional Midwives (CPMs) at home births (Johnson and Daviss, 2005b). Low birthweight or premature birth adds an average of $21,876 to the cost of caring for an infant (Russell et al., 2007), with additional health and financial repercussions later in life. If the number of births at home and in freestanding birth centers each increased by 5%, and the decrease in the populations served reflected the prematurity rates described above, we estimate that the reduced likelihood of low birthweight alone would contribute an additional savings of $111 million.

### Increased Competition for Hospitals

Competition is a moderating force for prices and an incentive for improved quality. Robinson (2011) found that hospitals with limited competition charged commercial insurers 13.0–25.1% more for specific procedures than hospitals in competitive markets. Again, CPMs can practice legally in only 36 states^11^. If legislation enables them to serve more of the 50 states and territories and join forces with the Certified Nurse-Midwives (CNMs) and Certified Midwives (CMs) who also attend births in homes and freestanding births centers, midwives can become low-cost, service-oriented hospital competitors.

The Big Push for Midwives is a national campaign in the US initiated and driven by consumers wanting to increase access to care by midwives attending births in the broader community, not just in the hospital. It focuses on increasing access to CPMs by pushing for legislation that legalizes them in the 14 holdout states and also on the need for CNMs to come out from the requirement of physician sign-off on their care:

We like to emphasize that competition is valued as an economic concept because it reduces costs and increases access and quality of goods and services for consumers. As the Big Push for Midwives Campaign posted on social media December 30, 2020,^12^ to the extent that public policy mandates hospitals or physicians to sign-off for a single visit, or that midwife-guidelines approval is granted to physicians, they have been handed the weapon they can use to limit the financial and clinical impact of competition. This is to provide clarification of the intent, and the possible negative effects, of organized medicine’s involvement in out-of-hospital midwife or birth center legislation^13^.

If stronger competition forced hospitals to reduce their price for an uncomplicated birth by 10%, the 3.51 million childbearers who would still deliver in the hospital under our scenario—or their insurers^14^—could save $4.267 billion. Because hospitals would still be the exclusive providers of care for complications, we assume here that only the price for an uncomplicated birth would decrease. There is substantial evidence that competition also affects treatment decisions in hospitals (Gaynor et al., 2015). Intensified competition from CPM-attended home births, which have a 5.2% cesarean rate (Cheyney et al., 2014), especially when accompanied by education for families about their options, should provide a financial incentive for hospitals to bring their cesarean rates within a more acceptable range (Again, the US national cesarean rate is 32%.) If US hospitals reduced cesareans to the 15% range, as the World Health Organization (WHO) has recommended since 1985, the savings for the birthing people who would still deliver in the hospital—and especially for their insurance companies--could be an additional $3.422 billion.

The total estimated savings from increased access to births outside the hospital as we have described above amount to $10.868 billion annually. This proposal to facilitate an increase in births at home or in freestanding birth centers, if implemented, would represent a huge win for the many constituents who want access to safe and normal physiologic childbirth with fewer interventions, freedom of choice for a variety of ideological, religious, cultural, financial or personal reasons, and lower maternity care costs for American society.

## Obstetric and Public Health Statements on Home Birth Prior to COVID-19

The successful implementation of US policy to increase rates of home and freestanding birth center births would be facilitated by at least tacit support from the national obstetric and public health communities. Some support has emerged: in 2001, the American Public Health Association (APHA) passed a resolution entitled, “Increasing Access to Out-Of-Hospital Maternity Care Services through State-Regulated and Nationally-Certified Direct-Entry Midwives,”(American Public Health Association, Maternal and Child Health Division, 2001) after they saw the methodology and preliminary data from the “CPM 2000” study on home births (Johnson and Daviss, 2005a).

A detailed description of the history and politics behind the American College of Obstetrics and Gynecology (ACOG) statements on home birth and a rationale for better integrating midwives specializing in births at home and in freestanding birth centers in the US can be found in Anderson et al. (2021). Briefly, ACOG officially opposed home birth from the 1970s on; 2011 was the first year that any evidence was quoted to support ACOG’s negative statements about it, but that evidence was based on part of a meta-analysis that was later discredited (Wax et al., 2010, analyzed in; Anderson et al., 2021). To their credit, ACOG removed the Wax et al. study from their equations about perinatal and neonatal mortality in the next ACOG statement on Planned Home Birth in 2016.

However, unfortunately, ACOG has not updated its analysis to include the two new home birth meta-analyses (Scarf et al., 2018; Hutton et al., 2019) that demonstrate no difference in safety among birth settings for low-risk childbearers. Instead, Table 2 in ACOG’s homebirth statements since 2016 has continued to use a single study based on birth certificates in a single state (Snowden et al., 2015) to assert that home birth “is associated with a more than twofold increased risk of perinatal death (1–2 in 1,000)^15^.” The analysis in Anderson et al. (2021) questions whether such a study can be generalized to other US. In short, the Snowden et al. study was conducted in Oregon, one of only two states where licensure was not required for midwives to practice legally at that time, and where family members, naturopaths, or unlicensed midwives managed more than a third of the births.

A subsequent interview published between the principal author of the study, Jonathan Snowden, and Melissa Cheyney, the midwife in the state who happened to be the principal author of the national homebirth study of the Midwives Alliance of North America (Cheyney et al., 2014) clarified that they had several common understandings: that the absolute risk of home birth in this and other studies is low; that the risk of having a cesarean in a planned hospital compared to planned home birth in Oregon and the rest of the US is dangerously high; that one should not assume that parents choose home birth for selfish reasons without taking their baby’s safety into consideration; and that better integration and respect for midwives in Oregon as well as the rest of the US could improve outcomes (Cheyney, 2016).

By 2016, with pressure from other obstetric associations and studies that could no longer be ignored, ACOG (ACOG, 2016) accepted that home birth does occur safely in other high-resource countries and that “a characteristic common to those cohort studies reporting comparable rates of perinatal mortality” among care settings is the provision of care by midwives “well integrated into the health care system.”

In their 2016–2020 statements (ACOG, 2016), ACOG also acknowledged that they would support the provision of care, not just by CNMs and CMs but by all midwives whose education and licensure meet the International Confederation of Midwives (ICM) Global Standards for Midwifery Education, which many CPMs do^16^.

The other two ACOG statements on birth setting since COVID-19 will be discussed in *Then COVID-19 Struck: Highlights Even More, Need for Legislation and Health Insurance for Birth Outside Hospitals*.

## What Evidence Do We Have About What Childbearers Want?

In the *Listening to Mothers* survey carried out by the California Health Care Foundation (2018), although 99% of women in the state had a hospital birth in 2016, a substantial portion expressed interest in using a freestanding birth center or their private home for a future birth. However, only 7% of women in California in the survey used midwives as their main prenatal care providers and 9% as their birth attendant:

Less than 1 in 10 survey participants used either midwives or labor doulas … for their recent births. However … over 1 in 6 women would definitely want midwives or labor doulas for a future birth. In addition, more than 1 in 3 would consider using these care team members^17^.

Some of this was the result of the lack of options of available insurance providers. For example, nearly 1 in 4 Black or Latina women had their prenatal care provider assigned to them, apparently by their primary provider, compared to less than 1 in 8 white women^17^.

**FIGURE 5:**
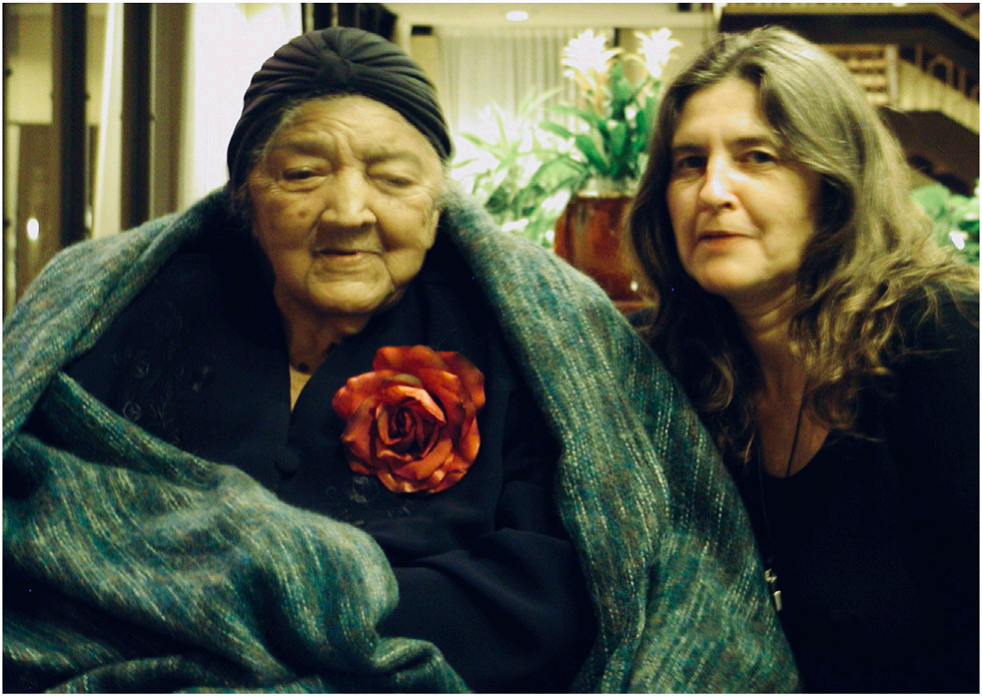
Visiting “Miss Margaret” Charles Smith, age 98, the year she died (2004). She attended circa 3500 babies at home in Alabama, many during times when African American women were denied entry to hospitals. Betty–Anne (on the right), who attended homebrths in Alabama 1979–81, studied the statistics at that time in Russell County, Alabama, trying to understand why the “Black granny midwives”–who decided they would rather be called, the “Grand Midwives”—were having their licences revoked. She discovered their outcomes were good, but a Medicaid pay hike for physicians and the 1982 introduction of nurse-midwives had made poor African American pregnant women financially lucrative for hospital practitioners (Financial Planning Division, Alabama Medicaid 1995). Interviewing the midwives and women, Betty-Anne realized that nobody had asked the women what *they* wanted. Photo by Ken Johnson. Used with permission.

**FIGURE 6:**
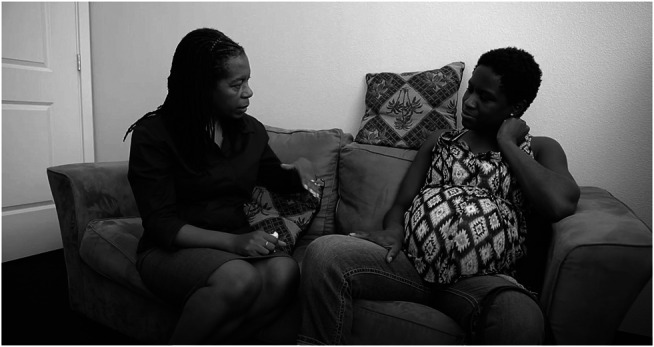
Midwives like Jennie Joseph (left), who practices in Florida, are picking up from where Miss Margaret and the other Grand Midwives of the South have left off -because the latter are no longer permitted to practice. However, even with her Certified Professional Midwife credential and state license, and in spite of the fact that she and her team have reduced prematurity and low birth weight rates within the Black, Indigenous, and People of Color community, their attempts to get any government support from grants or other public health or civic funds have been unsuccessful. She receives a meager fee of $1500 if clients are compensated through Medicaid, but even less for the over-proportion of indigent, undocumented and uninsured who aren′t on Medicaid who come to her freestanding birth center at “Commonsense Childbirth” in Orlando who receive care for free if needed, or on a sliding scale. Not supporting all pregnant women to have health care, during pregnancy or any other time of their life, is unheard of in countries like the UK where Jennie was originally trained as a midwife. These intimate moments of shared trust and respect, illustrated here between client Kristen April Brown (on the right) and Jennie, is what researchers have determined may be behind the consistently better outcomes compared to other clinics and services where women from the same demographic receive maternity care (Joseph 2021:131-144). Photo from “the American Dream,” videographer Paolo Patruno, see www.birthisadream.org and https://www.youtube.com/watch?v=Si_4xUQ2MK8&t=1s. Used with permission.

The financial impediment may explain some of why data from the National Vital Statistics database demonstrate that white women have 2 ½ times the rate of home births as American Indian or Alaskan Native women, three times the rate of Black women, and almost four times the rate of Hispanic women (Martin et al., 2019). (See Figures 4, 6, what Indigenous, Black and Latina women deserve to have offered, and Figure 5, how it was taken from them in the 1980s.)

The current President of the Midwives Alliance of North America, Sarita Bennett, emphasizes that there is a balancing place in US society for those not ready to choose birth in their own home but do not want to go to a hospital, especially during the pandemic:

While we can talk about legalizing CPMs, unless we also address changing birth center legislation that is restrictive rather than evidence-based, there will still be limited options, especially for those who might accept birth center birth but aren't ready to make the leap to home birth. My birth center in a state with no birth center legislation has lots of those families who then choose home birth the next time (Sarita Bennett DO, CPM, personal communication, Jan. 2021).

Pain relief is a major concern of birthing persons, may determine where they seek care, and is related to delivery cost. In the national Listening to Mothers survey of 2013, 67% of respondents used epidural or spinal analgesia, 16% used narcotics, and 7% were given general anesthesia^18^.

Some childbearers want to be more physically involved with their births and have fewer interventions. In the same survey, 17% said they used no pain medication, and 6% used nitrous oxide (the same “laughing gas” that dentists use), which is a client-controlled and effective method of pain relief and can be made available in birth centers and at home births. It is cheaper for birthing persons to use nitrous in home or birth centers, as hospitals can take advantage of the lack of regulation to charge what they want. For example, a hospital in Wisconsin bills more than $100 for every 15 minutes that the nitrous is sitting in the room, which, for one woman, resulted in a bill of $4,836, whereas the local freestanding birth center charges only a flat fee of $100 for its use, for as long as it is needed. An epidural in the same hospital in Wisconsin costs $1,500, a third of the price of the nitrous oxide^19^.

In the aforementioned 2013 *Listening to Mothers* national survey, women reported using a variety of drug-free methods to increase comfort and relieve pain, with 73% using at least one non-pharmacologic method of pain relief, led by breathing techniques (48%), position changes (40%), hands-on techniques like massage (22%), and mental strategies (e.g., relaxation methods) (21%)^18^.

## Then COVID-19 Struck: THE NEED FOR Legislation and Health Insurance for Birth Outside Hospitals BECOMES URGENT

A birthing person’s ability to pay for a birth in their private home or at a freestanding birth center is often limited by finances because most hospital births are paid for through public or private insurance, while births not in hospital are rarely afforded the same privilege. In 2017, more than 2/3 (67.9%) of planned home births and almost 1/3 (32.2%) of birth center births were paid for by the birthing persons themselves, while only 3.4% of women self-paid for hospital births (MacDorman and Declercq, 2019).

In 2020, the report Birth Settings in America: Outcomes, Quality, and Choice concluded:

Models for increasing access to birth settings for low-risk women that have been implemented at the state level include expanding Medicaid, Medicare, and commercial payer coverage to cover care provided at home and birth centers … by certified nurse midwives, certified midwives, and certified professional midwives whose education meets International Confederation of Midwives Global Standards … the potential impact of these state-level models is needed to inform consideration of nationwide expansion, particularly with regard to effects on reduction of racial/ethnic disparities in access, quality and outcomes of care [National Academies of Sciences, Engineering, and Medicine (NASEM) 2020:12]

Even prior to COVID-19, this report’s conclusions had drawn attention to the fact that there is a “mismatch” between the care needs of the population as a whole and what is available for them, in both rural and urban areas. The NASEM researchers concluded that for most childbearers, who are largely healthy, it is unnecessary to rely primarily on “a surgical specialty” (obstetrics) for frontline care. They pointed to a growing shortage of obstetricians due to job dissatisfaction and early retirement and to the next logical step—to use the already nationally credentialed midwives as primary care providers, as most other countries do. Furthermore, the report emphasizes a need to ensure that the workforce “resembles the racial/ethnic composition of the population … as well as its linguistic, geographic, and socioeconomic diversity,” because research demonstrates that such measures increase safety and satisfaction (National Academies of Sciences, 2020: 13). (See Figures 5 and 6)

Enter COVID-19. As the pandemic increased the demand for birth setting options, frustrations for childbearers wanting care in their homes also increased, as did the racial and socio-economic disparities between those who can and cannot afford choice of birth setting. Countries like Canada with universal health care coverage have removed this artificial financial barrier to home births and also established some freestanding birth centers, articulating the obvious—that births outside the hospital are cheaper and more welcoming than engagement with the hospital enterprise; almost all provincial Canadian governments now cover the birth wherever it occurs.

Canada provides a good example of how it is easier to adapt when pandemics or other challenging events occur if midwives are available who can offer a choice of birth settings^20^. Of the births being attended just by the midwives in Ontario (not the family docs or obstetricians), the planned home birth rate was 13% in March 2020, when the effects of COVID-19 were just beginning to be felt. By May 2020, with COVID-19 in full swing, the planned home birth rate among midwife-attended births in Ontario had increased from that 13–20% (Daviss et al., 2021). This increase was easily facilitated because all infrastructures—legislation, insurance coverage, quality assurance programs and integration—were already well established for homebirth providers. In March and April, clients who had formerly considered a hospital birth did not have to switch providers. They simply told their midwives that they now preferred to stay home.

The US states without adequate provisions for care at home or in freestanding birth centers even in normal times have been caught more unprepared than those that already had instituted providers for those birth options prior to COVID-19. Some jurisdictions like Washington, D.C^21^. and Kentucky^22^ managed to get legislation for CPMs passed just before the pandemic struck the US. Others (like Illinois, which has had a Home Birth Safety Act that would legalize CPMs on the books for about 10 years^23^) have remained sluggish at passing such legislation, in spite of obvious need (Ayres-Brown, 2020).

In New York, the strong need for increased access to births outside the hospital prompted Governor Cuomo’s Executive Order to invite midwives from outside the state of New York to come and help. This highlighted, and brought into question, the fact that in normal times, CPMs cannot legally practice there, just as they cannot in Illinois nor in the other states where they are not legal. In fact, CPMs living in New York have been persecuted for practicing rather than embraced in the state, even though the state has long allowed CNMs and CMs to attend home births (May and Davis-Floyd, 2006; Chamberlain, 2020). This is also despite the fact that New York CPMs would qualify for licenses if the state midwifery board had properly implemented the licensing statute that was approved by the state legislature in 1992^24^.

Vicki Hedley, Past-President of the Midwives Alliance of North America (MANA) and Senior Advisor to NYCPM—the New York State CPM organization—thinks that COVID-19 holds hope for change but explains the complications:

I do believe that this pandemic has potentially opened the door to legalization for CPMs in NY. More and more people are asking for our (CPM) services and wanting home birth because of the safety aspects. The problem is access. Although NY requires that licensed providers be paid by insurer’s reimbursements, many insurers require liability/malpractice insurance, which many home birth midwives cannot afford and more unfortunately cannot obtain due to the lack of state licensure. We are in a Catch-22. Straight Medicaid pays about $1,300 for [full-scope] maternity care, which is far from a living wage. Of course, these issues need to be addressed in order to create the access for birthing families that is so desperately needed (Personal communication, December 5, 2020).

Meanwhile, the temporary nature of the Governor’s Executive Order has caused serious problems for any CPM who does want to practice in the state to meet the increased demand by mothers and families for out-of-hospital birth options. Ida Darragh, the Executive Director of the North American Registry of Midwives (NARM), the organization responsible for setting standards for CPM credentialing nationally, describes the urgent need for legislation:

There is currently a proposal for licensure of CPMs in New York being drafted by the office of Dick Gottfried, the Chair of the Assembly Health Committee. It needs some better language before being submitted and the midwives are trying to communicate with the office about it. It is the optimum time to present a bill with several months of “legal” status during the pandemic already. The executive order is renewed monthly, but that means only that midwives with a license in another state can practice legally until that expiration date. Midwives and clients need more certainty than one month of legal status! (Personal communication December 5, 2020)

This ambiguous month-to-month situation puts the CPMs currently practicing in New York in a vulnerable state: being legal for a few months, but then with the potential to have their licensure removed just when their clients are actually due to have their babies!

ACOG and ACNM recognized early on that the pandemic had created an interest in home birth, alerting them to the fact that families were nervous about institutional birth settings. They issued a joint statement in March acknowledging the pandemic but assuring the public that “Hospitals and birth centers that are both licensed and accredited *remain safe places* to give birth in the United States^25^.” (italics added).

Three weeks later, on April 20, 2020, ACOG’s CEO issued a further statement:

ACOG and its members, in collaboration with the health care team, are dedicated to providing patient-centered, respectful care. Obstetrician-gynecologists see first hand the stress and uncertainty facing pregnant people, families, and their support networks during the COVID-19 pandemic, and this includes questioning the settings in which to give birth. However, even during this pandemic, *hospitals and accredited birth centers remain the safest places to give birth* [italics added]. Physicians, certified nurse-midwives and certified midwives, and the entire health care team will work to ensure that precautions are taken to make labor and delivery safe, supportive and welcoming for their patients (Phipps, 2020).

Earlier in the Phipps statement is the quote about the “more than twofold increased risk of perinatal death” of ACOG’s other statements over the last four years, which from the outset was rendered questionable, since the only source for such a claim in their Table on perinatal mortality is the single Oregon study of 2015, whose generalizability is doubtful for the other states (See *Obstetric and Public Health Statements on Home Birth Prior to COVID-19* above and Anderson et al., 2021). Instead, the states that legalize nationally certified midwives can benefit from cohort studies on midwives with like certification that demonstrate similar outcomes between home and hospital births (Murphy and Fullerton, 1998; Johnson and Daviss, 2005a; Stapleton et al., 2013).

Neither the ACOG nor the ACOG/ACNM statements provide any data to demonstrate that hospitals are now safe, safer, or “remain safer” than home births under COVID-19 pandemic conditions. As far as we know, there have been no data in the US comparing outcomes of different birth settings since COVID-19 began its surge across the country. There is, on the other hand, some data to indicate that it is reasonable for families to have concerns about entering the hospital if it is not necessary. Indeed, it is not necessary--in fact, may not be advisable–if you are a low risk birthing person.

Dr. Manoj Jain, an infectious disease specialist from Memphis, TN who recognized that a patient of his had likely acquired COVID-19 from staff (Jain, 2021) provides an example of what the academic literature has brought to light about possible infection in hospital. Front-line health care workers in the US have a three times greater risk of testing positive for COVID-19 than the general community (Nguyen et al., 2020). These providers can be highly contagious if they have COVID-19 themselves, prior to having any symptoms. While obstetricians, CNMs, and obstetric nurses are not usually considered front-line workers who deal with COVID-19 patients, they are walking in and out of the hospitals where COVID-19 patients gather, and, as the physician in the Memphis story points out, eat lunch without their masks on, with other health care workers, in the lounge or cafeteria.

The true wild cards in the hospital are the anesthesiologists and nurse anesthetists who, unlike obstetric providers, cannot limit where they work to one floor of the hospital. They don and doff—and sanitize--faithfully, but they may have to quickly move from an intubation on a COVID-19 patient in one ward to doing an epidural on a pregnant patient in another section of the hospital.

COVID-19 also adds a new dimension to avoiding the reality that ACOG has admitted: that there are increased cesarean births when low risk women choose hospital birth. Even if low risk women hope to be able to manage without an epidural, their likelihood of having a cesarean increases from 3.7% with a planned home birth to 19% if they plan a hospital birth (Johnson and Daviss, 2005a)^26^, which also increases their risk of exposure to more healthcare professionals in the operating room.

## Liability

Following the first large prospective home birth study that demonstrated similar safety between home and hospital births in North America (Johnson and Daviss, 2005a), out of thousands of responses to this study, the only response to the *British Medical Journal,* which published the study, from a practicing American physician iterated that he did “not mind” women choosing home birth, but that “our pernicious legal system prevents me from ever considering the practice” (Rivera, 2005).

The present liability system can create insurmountable financial risks for practitioners that make them reticent to offer valued services that childbearers are increasingly seeking. A team of researchers concerned about the impact of the present system identified seven aims for a high-functioning liability system and studied “whether 25 strategies that have been used or proposed for improvement have met or could meet the seven aims” (Sakala et al., 2013). They concluded:

Ten strategies seem to have potential to improve liability matters in maternity care across multiple aims. The most promising strategy--implementing rigorous maternity care quality improvement (QI) programs--has led to better quality and outcomes of care, and impressive declines in liability claims, payouts, and premium levels. A number of promising strategies warrant demonstration and evaluation at the level of states, health systems, or other appropriate entities. Rigorous QI programs have a growing track record of contributing to diverse aims of a high-functioning liability system and seem to be a win-win-win prevention strategy for childbearing families, maternity care providers, and payers. Effective strategies are also needed to assist families when women and newborns are injured.

COVID-19 raises new questions about liability for midwives who practice in private homes or freestanding birth centers. If there is a shortage of legal midwives based outside of hospital in any state, whether or not they are invited to temporarily practice as in New York state, or left without legal accommodation as in Illinois, midwives from neighboring states will inevitably come to the rescue of women in need in the state, regardless of their legal status (Ayers-Brown, 2020).

Even if midwives are legally attending births in private homes or freestanding births centers in any given state, if they don’t have hospital privileges, the increased restrictions of COVID-19 can have serious implications. Ida Darragh and Vicki Hedley explain that many hospitals are now allowing the father of the baby to attend the birth, and just recently in some places, a doula (often only if she is certified by the hospital or by an organization recognized by that hospital). However, when there is a transport from a home birth, the community midwife may not be able to enter the hospital along with her own client to provide the continuity of care that is so well proven in the literature to improve outcomes (Sandall et al., 2016). Thus important information that the midwife could provide can be missed--for example, the time of rupture of the membranes, the baby’s presentation, a borderline history of pre-eclampsia, or the special cultural and personal needs of a family. This could implicate both the midwife and the hospital in subsequent litigation.

Although legal reform is beyond the scope of this article, we would like to point out here that there are underutilized options to discuss and disseminate transfer and practice guidelines, to encourage swift and fair settlements in legal disputes (Anderson, 2003), and there are less litigious societies whose policies can serve as models, such as those of Sweden and Germany (Lowes, 2003).

## Conclusion: Expanded Access to Births in Private Homes and Freestanding Birth Centers in the US is Warranted

Home and birth center births are on the rise in the US, and COVID-19 has provided a catalyst/pivotal moment that directs us to the need for increased access to nationally credentialed, licensed midwives and options for women to birth outside the hospital. Many US women have already switched to these options to avoid both hospital contagion and the forced choice of only one (or no) personal birthing companion during these Covidian times.

As we have shown above, if only 10% more US women deliver at home or in freestanding birth centers, the savings could amount to $10.868 billion per year. Outcomes are similar for low-risk mothers regardless of setting in countries where midwives are well-trained and integrated into the Reproductive, Maternal, Newborn and Child Health (RMNCH) Continuum of Care in the community^27^. The US studies on birth settings demonstrate good and similar outcomes among home, birth center, and hospital births when: 1) they are based on charts for an identified cohort rather than on birth certificates; 2) they can identify low risk women; 3) they discern the planned place of birth, thereby avoiding counting accidental, unplanned out-of-hospital births; and 4) they have studied a defined group of midwives with training standards. Cost and safety issues suggest expanded access to home and freestanding birth centers as a solution to the shortage of appropriate services and maternity-care service providers that existed even before COVID-19.

Increased access to credentialed maternity-care providers requires new legislation for CPM licensure in some states and extended public insurance for home and freestanding birth center settings in all states. While the data on the safety of home and freestanding birth centers has convinced the APHA and many state legislatures over the last two decades to promote birth in these settings, COVID-19 and pure practicality have convinced more state politicians of the importance of credentialed and licensed midwives who offer these alternatives to hospital birth.

There are now two other important givens that mark change: First, ACOG has admitted that safe home birth is possible in other countries where midwives are well-integrated and in accredited birth centers in the US. Second, the New York State governor has invited licensed midwives, including CPMs from other states, to help out in his state during the pandemic (Executive Order, 2020), thereby recognizing their value and essential services in a state that has had former reserve towards CPMs.

Taking two critical further steps could integrate nationally credentialed midwives into the larger US health care system and help these midwives to meet demands of birthing people. The first is to build the infrastructure of legislation, insurance, and healthy Quality Improvement programs needed to support home, freestanding birth center, and hospital maternity care providers so they can be fully integrated into their local RMNCH Continuum of Care.

The second step is to encourage a culture in which all healthcare professionals recognize and encourage each other to offer the services for which they are best suited. This would include opening rather than limiting scope of practice, eliminating physician supervision but increasing collaboration, and encouraging autonomy of midwives and clients. It would also include debunking the myths of what is “safe” and “not safe.”

The first step is foreseeable and has been accomplished at least in part in about two-thirds of the United States. One would think it should be relatively easy, given the models in the other states, but of course it requires some buy-in to the second step. The second step is dependent on the first; in fact one might say the two steps are co-dependent. The second step requires visionary leaders who can turn over 100 years of conflict aside, expose the overlapping systems of self-protective competitors, and transmute the US maternity care system into a best-practice, safer and less costly model that puts the interests of the birthing population first.

Whether the primary goal is safety, reproductive justice, cost savings, avoiding infection, or increasing freedom of choice and access to birth options for birthing people, public policies that support planned, midwife-attended births in private homes and freestanding birth centers are the appropriate and long overdue response.

## Footnotes

2 For example, the Aetna insurance company states on its website that labor and delivery present “hazards” that “require standards for safety which are provided in the hospital setting and cannot be matched in the home situation” (Aetna, 2020).

3 This was an important recognition, as New York state has officially recognized only the Certified Nurse-Midwife (CNM) and Certified Midwife (CM) credentials. The CM credential is recognized in only 5 states and there are only around 120 practicing CMs, despite the fact that this credential was created by members of the American College of Nurse-Midwives (ACNM) in 1996. CMs go through the same training as CNMs (excluding the nursing component) and are certified by the same board. See May and Davis-Floyd (2006) for a full description of the creation of the CM and why it has not gone far. In contrast to the low numbers of CMs--which is also a direct-entry credential, there are around 3,000 CPMs practicing in the US.

4 Percentage-wise, though, the rate of homebirths in the Netherlands is much higher than in the US, currently standing at 13%, while that of the US stands at under 2%. The point is that the homebirth rate is rising in the US. In seven states in 2018 it was 2.0% or above—in Idaho, Montana, Oregon, Utah, Vermont, Washington, and Wisconsin (see Table I–5 in Martin et al., 2019 at https://www.cdc.gov/nchs/data/nvsr/nvsr68/nvsr68_13_tables-508.pdf).

5 In most US states, a non-CNM/CM midwife must first be a CPM to obtain a license, but some such midwives, once they have obtained licensure, drop their CPM certification rather than taking the trouble to renew it every 3 years.

6
http://narm.org/pdffiles/Statechart.pdf

7
https://www.georgiacpm.org/certified-professional-midwives-frequently-asked-questions

8 The “Continuum of Care” for reproductive, maternal, newborn and child health (RMNCH) includes integrated service delivery for mothers and children from pre-pregnancy to delivery, the immediate postnatal period, and childhood. Such care is provided by families and communities, through outpatient services, clinics and other health facilities…[It] recognizes that safe childbirth is critical to the health of both the woman and the newborn child—and that a healthy start in life is an essential step towards a sound childhood and a productive life (https://www.who.int/pmnch/about/continuum_of_care/en/).

9
https://www.pushformidwives.org/pushheadlines

10
https://birthmonopoly.com/covid-19/

11 PushMap and PushChart: https://www.pushformidwives.org/what_we_do

12
https://www.facebook.com/PushForMidwives/posts/3999886113363809

13
https://www.facebook.com/PushForMidwives/posts/3999886113363809 in response to https://newrepublic.com/article/160706/midwives-appalachia-kentucky-maternity-care-desert

14 In theory, it follows then, that if the insurers pay out less, they should be able to charge less.

15 In its 2017–2020 homebirth statements, the only changes that ACOG made from its 2016 statement were in Table 2 (a)the addition of another sign highlighted in yellow and explanation in the footnotes about what it meant: “includes planned birth center and home birth” and (b)the switching of signs (^‡^ and ^†^) that mark the Snowden et al. and Grunebaum et al. studies in the footnotes of Table 2. At first we thought they meant that the 3.9/1000 perinatal mortality figures were now being attributed to the Grunebaum study but we were mistaken. ACOG has continued to use the single study by Snowden et al. that reports 3.9/1000 perinatal deaths for planned home vs. 1.8/1000 perinatal deaths for hospital births (a “more than twofold risk”) for the reporting of perinatal mortality in its statements from 2016 to 2020.

16 The complexities of which CPMs do and do not meet these ICM standards are too detailed to explain herein. For the standards themselves, see https://internationalmidwives.org/assets/files/general-files/2018/04/icm-standards-guidelines_ammended2013.pdf

17
https://www.chcf.org/wp-content/uploads/2018/08/ListeningMothersCareTeam2018.pdf

18
https://www.ncbi.nlm.nih.gov/pmc/articles/PMC3894594/pdf/JPE23-1_PTR_A3_009-016.pdf

19
https://www.npr.org/sections/health-shots/2019/05/28/726572880/bill-of-the-month-4-836-charge-for-laughing-gas-during-childbirth-is-no-joke

20 For examples of effective care in the immediate aftermaths of earthquakes, tsunamis and floods, see Davis-Floyd et al., 2021; Lim and Davis-Floyd, 2021

21
https://code.dccouncil.us/dc/council/laws/23-97.html. Accessed December 17, 2020.

22
https://newrepublic.com/article/160706/midwives-appalachia-kentucky-maternity-care-desert

23
https://www.ilga.gov/legislation/BillStatus.asp?DocNum=1754&GAID=14&DocTypeID=SB&LegID=104736&SessionID=91&SpecSess=&Session=&GA=100

24 PushMap and PushChart: https://www.pushformidwives.org/what_we_do

25
https://www.acog.org/news/news-releases/2020/03/patient-centered-care-for-pregnant-patients-during-the-covid-19-pandemic

26 The cesarean rate is 5.2% overall in the more recent study (Cheyney et al., 2014) but it was difficult to find the rate among low risk women in hospital for a comparison to the study. In our 2005 report we were able to obtain it.

27
https://d3n8a8pro7vhmx.cloudfront.net/pushformidwives/pages/1144/attachments/original/1585429341/The_Big_Push_for_Midwives_Campaign_Strategic_Priorities.pdf?1585429341
